# Effects of GLP-1 Receptor Agonists on Psoriasis: An “Agent-Specific” Systematic Review of the Literature

**DOI:** 10.3390/jcm15135126

**Published:** 2026-07-01

**Authors:** Andrea Marani, Eleonora Neri, Edvige Morea, Davide Bertolla, Giulio Gualdi, Alessandro Borghi, Andrea Conti, Paolo Amerio

**Affiliations:** 1 Dermatologic Unit, Department of Surgery, AUSL Romagna, 47923 Rimini, Italy; 2 Section of Dermatology and Infectious Diseases, Department of Medical Sciences, University of Ferrara, 44124 Ferrara, Italy; 3 Clinic of Dermatology, Department of Medicine and Aging Sciences, University G. d’Annunzio, 66100 Chieti, Italy; 4 Dermatology Unit, Department of Medicine and Surgery, University of Parma, 43126 Parma, Italy

**Keywords:** GLP1RA, psoriasis, semaglutide, liraglutide, exenatide, tirzepatide, obesity, metabolic syndrome, diabetes

## Abstract

**Background:** Psoriasis is a chronic inflammatory disease frequently associated with obesity and type 2 diabetes mellitus (T2DM). Glucagon-like peptide-1 receptor agonists (GLP-1RAs), widely used for T2DM and obesity, have demonstrated anti-inflammatory and immunomodulatory properties that may be relevant in psoriasis. However, individual GLP-1RAs differ substantially in their pharmacological characteristics and clinical effects. Our objective was to systematically review the available evidence on the effects of individual GLP-1RAs in patients with psoriasis. **Methods:** A systematic review was conducted according to PRISMA 2020 guidelines. PubMed and Scopus were searched up to April 2026 for studies evaluating GLP-1RAs in psoriasis. Case reports, case series, observational studies, and randomized controlled trials were included. Preclinical, clinical and safety outcomes were extracted and narratively synthesized. **Results:** Twenty-six studies met the inclusion criteria. Most involved patients with concomitant obesity and/or T2DM. Overall, semaglutide, liraglutide, exenatide, and tirzepatide were associated with improvements in psoriasis severity, often accompanied by reductions in body weight, glycated haemoglobin, inflammatory markers, and cardiometabolic risk factors. Semaglutide and liraglutide showed the most consistent evidence of benefit. Experimental and clinical data also suggested direct immunomodulatory effects on pathways involved in psoriasis pathogenesis. However, paradoxical psoriasiform eruptions and psoriasis exacerbations were reported with some agents. The evidence base was limited by the predominance of case reports and small observational studies, substantial heterogeneity, and the limited availability of randomized controlled trials. **Conclusions:** Current evidence suggests that GLP-1RAs may improve both psoriatic disease activity and cardiometabolic outcomes, particularly in patients with obesity or T2DM. Nevertheless, potential differences among individual agents warrant further investigation in larger controlled studies.

## 1. Introduction

Psoriasis is a chronic immune-mediated inflammatory disease affecting approximately 1–3% of the global population with considerable geographic variability in prevalence [1]. Clinically, it is characterized by well-demarcated erythematous plaques covered with silvery scales, most commonly involving the extensor surfaces, scalp, and lumbosacral region, although any skin site may be affected [2]. Psoriasis can present in several phenotypic variants, with chronic plaque psoriasis accounting for nearly 90% of cases, and may be associated with nail involvement and psoriatic arthritis, which affects up to 25% of patients [2]. Historically considered a disorder limited to the skin, psoriasis is now widely recognized as a systemic inflammatory disease, often referred to as “psoriatic disease” [3]. This broader concept reflects the frequent coexistence of multiple comorbidities, including obesity, type 2 diabetes mellitus (T2DM), metabolic syndrome, cardiovascular disease, and depression, all of which share common inflammatory pathways [1,3]. The recognition of psoriasis as a systemic condition has important therapeutic implications, highlighting the need for treatments capable of targeting both cutaneous manifestations and underlying metabolic and inflammatory dysregulation. From a pathogenic perspective, psoriasis results from a complex interplay between genetic predisposition, environmental triggers, and immune system dysregulation. Central to disease development is the activation of dendritic cells and the subsequent stimulation of the interleukin (IL)-23/Th17 axis, leading to the production of pro-inflammatory cytokines such as IL-17, IL-23, and tumor necrosis factor-α (TNF-α) [3]. These mediators promote keratinocyte hyperproliferation, impaired differentiation, and sustained recruitment of inflammatory cells within the skin, ultimately resulting in the formation of characteristic psoriatic plaques [2]. In addition, systemic inflammation is further amplified by metabolic factors, particularly in patients with obesity and T2DM, where adipokine imbalance and chronic low-grade inflammation contribute to disease severity [3]. Therefore, obesity and T2DM are not merely common comorbidities in psoriasis, but active contributors to systemic inflammation through adipokine dysregulation and metabolic dysfunction [4,5,6]. Adipose tissue itself acts as an immunologically active organ capable of producing pro-inflammatory cytokines and adipokines, thereby contributing to chronic low-grade systemic inflammation and amplification of psoriatic disease activity [4,6,7]. In recent years, increasing attention has consequently been directed toward metabolic-targeted therapies capable of modulating both metabolic dysfunction and inflammatory pathways involved in psoriatic disease.

Glucagon-like peptide-1 (GLP-1) is an incretin hormone physiologically secreted by enteroendocrine L-cells of the small intestine in response to nutrient intake. GLP-1 enhances glucose-dependent insulin secretion, suppresses glucagon release, delays gastric emptying, and promotes satiety, thereby contributing to glycemic regulation and body weight control [5,8]. Glucagon-like peptide-1 receptor agonists (GLP-1RAs) are pharmacological analogues of endogenous GLP-1 originally developed for the treatment of T2DM and, more recently, obesity. Table 1 shows currently marketed and emerging incretin-based therapies while Table 2 shows pharmacological characteristics of GLP-1Ras included in this review. Clinical trials show the superiority of GLP-1RAs to other antihyperglycemic drugs in reaching glycemic targets, reducing weight and blood pressure, and having a cardioprotective effect, without the risk of hypoglycemia. These drugs have transformed the guidelines for the management of diabetes [9]. These agents mimic the biological effects of endogenous GLP-1 while exhibiting longer half-lives and greater pharmacological stability than native GLP-1 itself [5,10]. Beyond glycemic control and weight reduction, these agents have demonstrated important cardiovascular, anti-inflammatory, and immunomodulatory properties [7,11]. Increasing evidence suggests that GLP-1RAs may influence psoriasis through both indirect metabolic effects and direct immunomodulatory mechanisms. From a metabolic perspective, weight reduction and improvement of adipose-tissue dysfunction may decrease the production of pro-inflammatory adipokines involved in systemic and cutaneous inflammation, thereby indirectly improving skin condition [6,12]. Semaglutide has been associated with significant reductions in inflammatory biomarkers, visceral adiposity, epicardial adipose tissue inflammation, and cardiometabolic risk parameters, potentially contributing to reduction in systemic inflammatory burden [12]. In large cardiovascular outcome trials, semaglutide significantly reduced major adverse cardiovascular events independently of baseline BMI and weight loss [13]. In parallel, experimental and clinical studies suggest that GLP-1RAs may directly modulate inflammatory pathways involved in psoriasis pathogenesis. GLP-1 receptors have been identified in psoriatic plaques but not in unaffected skin, suggesting a potential role in lesional immune responses [14]. Furthermore, GLP-1RA therapy has been associated with modulation of key inflammatory mediators and signalling pathways implicated in psoriatic disease, including IL-17, IL-23, TNF-α, IL-6, NF-κB, and AMPK, as well as immune-cell trafficking [4,5,7]. Liraglutide has been associated with modulation of invariant natural killer T (iNKT) cell trafficking, suppression of IL-23/Th17 signalling, and reduction in dermal inflammatory infiltration in psoriatic models [14,15,16].

At the same time, although most available evidence suggests a beneficial effect of GLP-1RAs in psoriasis, paradoxical and adverse cutaneous reactions have also been reported. Case reports and pharmacovigilance analyses have documented psoriasiform eruptions and psoriasis exacerbations associated with GLP-1-based therapies, highlighting the complexity of their immunological effects and the need for further investigation [18,19,20]. Although GLP-1RAs are generally considered a single therapeutic class, individual agents differ in pharmacokinetic characteristics, metabolic potency, and available clinical evidence [7,9]. These differences may influence both metabolic and immunological outcomes and could therefore be relevant in psoriasis. Consequently, the dermatological effects observed in psoriasis may not be entirely homogeneous across the GLP-1RA class, and specific agents could theoretically provide distinct therapeutic advantages according to the metabolic and inflammatory profile of individual patients.

Therefore, a “molecule-specific” analysis is needed to explore whether the dermatological effects observed in psoriasis are shared by the entire GLP-1RA class or whether specific agents may show a more favourable profile in terms of cutaneous improvement, systemic benefit, tolerability, or paradoxical cutaneous reactions. For this reason, the present systematic review aims to clarify the role of GLP-receptor agonists in psoriatic disease, both in terms of therapeutic benefit and potential adverse or paradoxical effects, evaluating both the overall impact of GLP-1-based therapies as a pharmacological class and the evidence available for each individual agent, in order to investigate whether specific drugs may appear more suitable as adjunctive therapeutic options in patients with psoriasis and metabolic comorbidities.

## 2. Materials and Methods

This systematic review was conducted according to the Preferred Reporting Items for Systematic Reviews and Meta-Analyses (PRISMA) 2020 guidelines [21].

### 2.1. Data Sources and Search Strategy

Previous reviews investigating the role of glucagon-like peptide-1 receptor agonists (GLP-1RAs) in psoriasis were identified and screened, including the updated systematic review and meta-analysis by Ku et al. and the narrative review by Atiquzzaman et al. [4,16]. Primary studies included in these reviews were retrieved and reassessed in full text. A systematic literature search was conducted using PubMed and Scopus to identify studies evaluating the effects of GLP-1RAs in patients with psoriasis. The search strategy included combinations of the following keywords and Medical Subject Headings (MeSH) terms: PubMed: Semaglutide[tiab] AND psoriasis[tiab]; liraglutide[tiab] AND psoriasis[tiab]; dulaglutide[tiab] AND psoriasis[tiab]; exenatide[tiab] AND psoriasis[tiab]; lixisenatide[tiab] AND psoriasis[tiab]; tirzepatide[tiab] AND psoriasis[tiab].

Scopus: TITLE-ABS-KEY (semaglutide) AND TITLE-ABS-KEY (psoriasis); TITLE-ABS-KEY (liraglutide) AND TITLE-ABS-KEY (psoriasis); TITLE-ABS-KEY (dulaglutide) AND TITLE-ABS-KEY (psoriasis); TITLE-ABS-KEY (tirzepatide) AND TITLE-ABS-KEY (psoriasis); TITLE-ABS-KEY (lixisenatide) AND TITLE-ABS-KEY (psoriasis); TITLE-ABS-KEY (exenatide) AND TITLE-ABS-KEY (psoriasis).

Studies published up to April 2026 were considered eligible for inclusion. In addition, reference lists of relevant reviews and eligible studies were manually screened (citation searching) to identify potentially relevant publications published after completion of the electronic database search. Only articles published in English were included. No restrictions regarding study design were applied, and case reports, case series, prospective cohort studies, retrospective studies, randomized controlled trials, and observational studies were considered eligible for inclusion. Studies were included if they reported clinical, metabolic, immunological, or cardiovascular outcomes associated with GLP-1RA therapy in patients with psoriasis or psoriatic disease. Studies describing paradoxical psoriasis reactions or psoriasis onset during GLP-1RA therapy were also included. Review articles, conference abstracts without sufficient clinical data, expert opinions, editorials, and studies not specifically evaluating psoriasis-related outcomes were excluded. Titles and abstracts were independently screened to assess eligibility. Full texts of potentially relevant articles were subsequently reviewed. Extracted data included study design, patient characteristics, psoriasis subtype, metabolic comorbidities, previous and concomitant psoriasis treatments, GLP-1RA regimen, duration of follow-up, psoriasis outcomes, metabolic outcomes, and adverse events. No eligible studies evaluating the effects of lixisenatide or dulaglutide on psoriasis were identified. Records retrieved through the corresponding search strategies were either excluded after full-text review or focused on other GLP-1 receptor agonists without providing psoriasis-specific outcomes related to lixisenatide or dulaglutide. Consequently, no studies involving these agents were included in the data tables.

### 2.2. Selection of Studies

**Figure 1:**
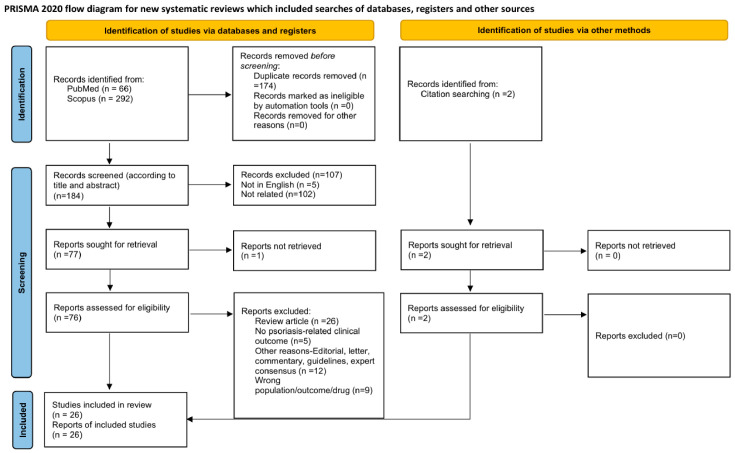
Flow chart describing the selection process for the literature reviewed.

The study selection process was conducted in accordance with the PRISMA 2020 flow chart (Figure 1). The electronic database search yielded a total of 358 records (PubMed, *n* = 66; Scopus, *n* = 292). After removal of duplicate records, the remaining articles were screened by title and abstract, and potentially relevant reports were examined in full text to determine eligibility. Articles were selected for this review if they reported clinical, metabolic, immunological, cardiovascular, or adverse dermatological outcomes associated with GLP-1 receptor agonist therapy in patients with psoriasis or psoriatic disease and were published in English. Full-text articles of the selected studies were retrieved and assessed. Articles meeting the eligibility criteria were included in the final analysis, together with those originally included in the updated systematic review and meta-analysis by Ku et al. and the narrative review by Atiquzzaman et al. [4,16]. In addition, the reference lists of relevant reviews and eligible studies were manually screened (citation searching) to identify potentially relevant publications not retrieved through the electronic database search. Two additional studies were identified through this process and were included in the final analysis.

### 2.3. Data Extraction

The data extraction process is shown in Figure 1.

### 2.4. Data Synthesis

Considering the substantial methodological and clinical heterogeneity among the included studies, which comprise case reports, case series, observational studies, and randomized clinical trials, a quantitative meta-analysis was not considered appropriate. The findings were therefore synthesized narratively and organized according to the specific GLP-1 receptor agonist evaluated [Semaglutide, Exenatide, Tirzepatide and Liraglutide]. Clinical outcomes, including psoriasis severity index (e.g., PASI), metabolic parameters, and adverse events, were extracted and summarized descriptively. Particular attention was paid to differences in study design, patient characteristics, concomitant treatments, follow-up duration, and outcome reporting.

### 2.5. Quality Assessment and Risk of Bias

The methodological quality and risk of bias of the included studies were assessed according to study design. Case reports and case series were evaluated using the Joanna Briggs Institute (JBI) Critical Appraisal Checklists for Case Reports and Case Series, respectively. Observational cohort studies were assessed using the Newcastle–Ottawa Scale (NOS), whereas Randomized Clinical Trials were evaluated using the revised Cochrane Risk of Bias tool (RoB 2). The results of the quality assessment are reported in Supplementary Table S1.

### 2.6. Protocol Registration

No review protocol was prospectively registered for this study.

### 2.7. Use of Generative Artificial Intelligence

Generative artificial intelligence (GenAI) (ChatGPT GPT-5.2 Instant, OpenAI, San Francisco, CA, USA) was used exclusively to assist with language editing, text refinement, formatting, and manuscript organization. All literature searches, study selection, data extraction, data analysis, interpretation of findings, and final scientific content were performed and verified by the authors.

## 3. Results

A total of 26 studies were included in this review, comprising case reports, case series, prospective cohort studies, randomized clinical trials, and large population-based cohort studies. Most studies evaluated patients with concomitant obesity and/or T2DM. The majority of available evidence consisted of observational studies and isolated clinical reports, whereas randomized controlled evidence remained limited. Included studies investigated both beneficial dermatological outcomes and paradoxical psoriasis reactions associated with GLP-1RA therapy.

## 4. Semaglutide

Among the studies evaluating GLP-1RAs in psoriasis, 5 publications specifically investigated the effects of semaglutide on psoriatic disease activity and associated metabolic parameters. These studies included case reports, a prospective cohort study, and an open-label randomized clinical trial, for a total of 61 patients treated with semaglutide-based regimens [12,18,22,23,24]. The majority of patients presented with moderate-to-severe chronic plaque psoriasis, frequently associated with obesity, T2DM, and other metabolic comorbidities, including metabolic dysfunction-associated steatotic liver disease (MASLD). Several patients had longstanding or treatment-refractory disease that was poorly responsive to previous therapies, including topical corticosteroids, acitretin, and biologic agents such as adalimumab [22,24]. Baseline psoriasis severity varied considerably across studies, with PASI scores ranging from approximately 10.6 [18] to 33.2 [22]. In the included studies, semaglutide was generally administered subcutaneously using gradual dose-escalation protocols to improve tolerability. Most regimens were initiated at 0.25 mg/week and progressively increased to maintenance doses ranging from 1 mg/week to 2.4 mg/week, depending on treatment indication and study design [12,18,22,24], while in the randomized clinical trial conducted by Petković-Dabić et al., semaglutide was administered at a fixed dose of 1.0 mg subcutaneously once weekly [23]. Follow-up duration ranged from 24 weeks to 10 months, although most studies evaluated outcomes after approximately 6 months of therapy. The results of our systematic review on semaglutide are set out in Table 3.

Detailed study characteristics, metabolic outcomes and non-PASI clinical outcomes reported in semaglutide-treated patients with psoriasis are provided in Supplementary Table S2.

The effect of semaglutide on PASI is shown in Table 3A.

### 4.1. Clinical Outcomes

Overall, semaglutide therapy was consistently associated with substantial dermatological and metabolic improvement across the included studies. Marked reductions in psoriasis severity were observed both in isolated case reports and in prospective observational and interventional studies, frequently accompanied by improvement in metabolic and inflammatory parameters [12,18,22,23,24]. In severe or treatment-refractory psoriasis, PASI reductions ranged from approximately 48% to over 90%, with some patients achieving near-complete clinical clearance after semaglutide initiation. Costanzo et al. reported a reduction in PASI from 33.3 to 2.6 after 10 months of treatment, corresponding to a 92% reduction, together with improvement in DLQI from 26 to 0 [22]. Similarly, Malavazos et al. described marked improvement in both PASI (12 → 0.2) and DLQI (20 → 1) during a 10-month follow-up period [12]. Comparable findings were reported by Lin et al., in whom PASI decreased from 22 to 6.2 and DLQI from 27 to 8 after 24 weeks of semaglutide therapy [24]. In the prospective cohort study conducted by Nicolau et al., PASI decreased from 10.6 to 5.5, corresponding to an approximately 48% reduction, while DLQI improved from 11.7 to 5 [18].

More robust evidence was provided by the open-label randomized clinical trial conducted by Petković-Dabić et al., evaluating semaglutide in obese patients with psoriasis and T2DM receiving chronic metformin therapy. Semaglutide treatment resulted in significant improvement in PASI and DLQI scores, with thirteen patients achieving PASI90 and one patient achieving PASI100 [23]. Beyond cutaneous improvement, semaglutide therapy was consistently associated with favourable metabolic effects. Significant reductions in BMI, visceral adiposity, glycated haemoglobin, LDL cholesterol, CRP levels, and inflammatory biomarkers including IL-6 were reported across multiple studies [12,18,23,24]. Malavazos et al. reported a reduction in BMI from 30.4 kg/m^2^ to 22.6 kg/m^2^ and normalization of glycated haemoglobin from 6.5% to 5.1%, together with reductions in epicardial adipose tissue (EAT) and pericoronary adipose tissue (PCAT) attenuation values after treatment [12]. Lin et al. additionally observed BMI reduction from 26.18 kg/m^2^ to 24 kg/m^2^ and normalization of liver enzyme values during follow-up [24]. In the study conducted by Petković-Dabić et al., semaglutide treatment was associated with reductions in IL-6 (3.5 pg/mL → 2.8 pg/mL), CRP (3.8 mg/L → 1.9 mg/L), LDL cholesterol (3.6 ± 1.1 mmol/L → 2.8 ± 0.9 mmol/L), and BMI (35.04 ± 5.9 kg/m^2^ → 30.7 ± 3.8 kg/m^2^) [23]. Nicolau et al. additionally demonstrated reductions in subcutaneous adipose tissue, preperitoneal fat, CRP levels, and Beck Depression Inventory (BDI) scores following treatment [18]. Most patients included in semaglutide studies had concomitant obesity, T2DM, or other metabolic comorbidities, reinforcing the close relationship between psoriasis severity and metabolic dysfunction. Although several patients had previously failed topical agents or biologic therapies before semaglutide initiation, concomitant biologic therapies specifically maintained during semaglutide treatment were not consistently reported [22,24].

### 4.2. Safety Profile and Adverse Reactions

Overall, semaglutide was generally well tolerated in the studies included. The most commonly reported adverse events were mild gastrointestinal symptoms, particularly nausea during dose escalation [18,23,24]. No paradoxical psoriasis exacerbations specifically attributed to semaglutide were identified among the included reports. No concomitant biologic therapies specifically maintained during semaglutide treatment were clearly reported in the included studies. Most patients had previously failed topical agents or biologic therapies before GLP-1RA initiation [12,18,22,24].

## 5. Exenatide

Among the studies evaluating GLP-1RAs in psoriasis, evidence regarding exenatide remained limited and primarily derived from isolated case reports and translational studies involving patients with concomitant obesity and/or T2DM, for a total of 3 patients treated with an exenatide-based regimen [14,26,27]. Overall, the available studies investigated both beneficial dermatological effects and paradoxical inflammatory reactions associated with exenatide therapy. Two of the reported patients presented with chronic plaque psoriasis, associated with longstanding metabolic comorbidities and previous failure of conventional psoriasis therapies [14,27]. Baseline psoriasis severity ranged from moderate disease (PASI 11) [27] to over 15 [14]. No concomitant biologic therapy was reported during exenatide treatment [14,26,27]. Exenatide was administered subcutaneously in most of the included studies [14,26,27]. Therapeutic regimens consisted of 5 μg twice daily [14,26,27]. Follow-up duration ranged from a few days in paradoxical reaction reports to approximately 12 months in studies evaluating clinical improvement [27]. Despite the predominantly favourable findings, paradoxical psoriasiform reactions associated with exenatide were also described. Bostan et al. reported the development of widespread pruritic psoriasiform dermatitis four days after exenatide initiation in a 56-year-old woman with T2DM. Histopathological examination demonstrated parakeratosis, acanthosis, hypogranulosis, and neutrophilic infiltration, while clinical improvement was observed after exenatide discontinuation [26]. The results of our systematic review on exenatide are set out in Table 4A,B.

Detailed study characteristics, metabolic outcomes and non-PASI clinical outcomes reported in exenatide-treated patients with psoriasis are provided in Supplementary Table S3.

The effect of exenatide on PASI is shown in Table 4C.

### 5.1. Clinical Outcomes

The available evidence generally supports a beneficial effect of exenatide on psoriasis severity and metabolic control. One of the earliest reports describing improvement of psoriasis during GLP-1RA therapy was published by Buysschaert et al. in 2012, describing a patient with longstanding extensive psoriasis and T2DM who experienced rapid dermatological improvement shortly after exenatide initiation [27]. During follow-up, PASI decreased from 11 to approximately 3–4 within the first month of therapy. Importantly, discontinuation of exenatide was associated with worsening glycaemic control, progressive weight regain, and psoriasis relapse, with PASI increasing to values exceeding 10. Reintroduction of exenatide again resulted in rapid clinical improvement, with PASI subsequently decreasing to 3.1 after one year of treatment, together with improved metabolic parameters and weight reduction [27]. Notably, the patient was not receiving concomitant biologic therapy during exenatide treatment.

### 5.2. Safety Profile and Adverse Reactions

Although exenatide was associated with clinical improvement in some patients [14,15,27], paradoxical psoriasiform reactions were also reported. Bostan et al. described a 56-year-old woman with T2DM who developed widespread pruritic psoriasiform dermatitis 4 days after initiation of exenatide therapy (2 × 5 μg/day) [26].

## 6. Tirzepatide

Evidence regarding tirzepatide remains limited but is rapidly expanding. Early evidence consisted mainly of case reports and small case-series analyses involving a total of 12 patients with psoriasis treated with tirzepatide-based regimens [28,29,30], whereas more recent phase 3b randomized clinical trials have substantially expanded the available evidence base [31,32]. Most reported patients presented with concomitant obesity and metabolic dysfunction [28,29], reflecting the close association between psoriasis severity and cardiometabolic comorbidities.

In the case series conducted by Gisondi et al., all patients had moderate-to-severe plaque psoriasis receiving stable biologic therapy for at least 12 months before tirzepatide initiation, including secukinumab, adalimumab, ustekinumab, guselkumab, and tildrakizumab [29].

Tirzepatide was generally administered subcutaneously, using gradual dose-escalation protocols to improve tolerability. Gisondi et al. described a Tirzepatide-based regimen which consisted of an initial administration of 2.5 mg/week, subsequently escalated to 5 mg/week [29], while Cook and Selby described a severe paradoxical eruption following escalation [30]. Follow-up was approximately 6 months, both in studies evaluating clinical improvement and in those describing paradoxical [28,29,30].

Recent evidence from dedicated phase 3b randomized clinical trials has substantially expanded the available evidence regarding tirzepatide in psoriatic disease. Lebwohl et al. conducted the phase 3b TOGETHER-PsO trial, in which 274 adults with moderate-to-severe plaque psoriasis and overweight or obesity were randomized to receive ixekizumab together with tirzepatide or ixekizumab alone. At week 36, combination therapy resulted in significantly higher rates of simultaneous PASI100 achievement and ≥10% weight loss (27.1% vs. 5.8%; *p* < 0.001), as well as higher PASI100 response rates (40.6% vs. 29.0%; *p* = 0.04). Significant improvements were also observed in quality of life and cardiometabolic outcomes, including body weight, blood pressure, lipid profile, glycated haemoglobin level [31]. Merola et al. subsequently reported the results of the TOGETHER-PsA trial, which demonstrated that the addition of tirzepatide to ixekizumab significantly improved disease activity outcomes, physical function, quality of life, and weight reduction in patients with active psoriatic arthritis and overweight or obesity. The primary endpoint, defined as the simultaneous achievement of ACR50 and ≥10% weight loss, was achieved by 31.7% of patients receiving combination therapy compared with 0.8% of those receiving ixekizumab alone (*p* < 0.001). Significant improvements were also observed in skin outcomes and multiple patient-reported measures [32]. Collectively, these studies provide the first randomized clinical evidence supporting a therapeutic strategy that simultaneously targets inflammatory disease activity and obesity-related metabolic dysfunction in patients with psoriatic disease. Nevertheless, these findings should be interpreted cautiously, as both trials evaluated tirzepatide in combination with ixekizumab rather than as monotherapy, making it difficult to isolate the specific contribution of tirzepatide to the observed dermatological outcomes.

The results of our systematic review on tirzepatide are set out in Table 5A,B.

Detailed study characteristics, metabolic outcomes and non-PASI clinical outcomes reported in tirzepatide-treated patients with psoriasis are provided in Supplementary Table S4.

The effect of tirzepatide on PASI is shown in Table 5C.

### 6.1. Clinical Outcomes

Gisondi et al. evaluated tirzepatide therapy (2.5 mg/week for 1 month, escalated to 5 mg/week for 5 months) in a case series of 10 obese patients with moderate-to-severe plaque psoriasis receiving stable biologic therapy for at least 12 months. At month six, mean PASI decreased from 6.4 ± 0.68 to 0.3 ± 0.75, corresponding to a 95% reduction, while DLQI improved by 81%. Significant metabolic improvements were additionally observed, including reductions in body weight (107.2 ± 7 kg → 94.1 ± 5.9 kg), waist circumference (116 ± 7 cm → 104 ± 6 cm), LDL (104 ± 11 mg/dL → 98 ± 10 mg/dL), triglycerides (181 ± 23 mg/dL → 146 ± 19 mg/dL), and glycaemia (96 ± 6 mg/d. → 94 ± 6 mg/dL) [29]. Mild nausea and intermittent diarrhoea represented the most frequently reported adverse events. More robust evidence was subsequently provided by the phase 3b TOGETHER-PsO trial conducted by Lebwohl et al., in which 274 adults with moderate-to-severe plaque psoriasis and overweight or obesity received tirzepatide in combination with ixekizumab. At week 36, combination therapy resulted in significantly higher rates of simultaneous PASI100 achievement and ≥10% weight loss compared with ixekizumab alone (27.1% vs. 5.8%; *p* < 0.001), together with higher PASI100 response rates (40.6% vs. 29.0%; *p* = 0.04). Significant improvements were also observed in quality of life and cardiometabolic parameters, including body weight, blood pressure, lipid profile, and glycated haemoglobin levels [31].

### 6.2. Safety Profile and Adverse Reactions

In contrast to the beneficial effects observed by Gisondi et al. [29], paradoxical psoriasiform eruptions and psoriasis flares associated with tirzepatide have also been described. Neptune Rosa et al. reported the case of a 37-year-old woman with previously stable plaque psoriasis in remission who developed a generalized psoriasis flare approximately four weeks after tirzepatide initiation for weight loss. The eruption progressively involved the trunk and extremities despite topical corticosteroid therapy. Tirzepatide discontinuation together with escalation of systemic psoriasis therapy resulted in gradual clinical improvement [28]. Similarly, Cook and Selby described a 59-year-old woman with obesity who developed severe guttate and inverse psoriasiform eruption involving approximately 40% body surface area after tirzepatide therapy. The patient had no personal or family history of psoriasis. Approximately three months after dose escalation, the patient developed rapidly progressive erythematous and desquamative plaques involving intertriginous areas, trunk, scalp, and extremities, associated with marked pruritus. Histopathological examination demonstrated psoriasiform epidermal hyperplasia with parakeratosis and neutrophilic infiltration. Tirzepatide withdrawal together with initiation of systemic dermatologic treatment (ixekizumab and later risankizumab because of the severity of the cutaneous eruption) resulted in progressive clinical improvement [30]. Overall, the available evidence regarding tirzepatide demonstrated heterogeneous dermatological outcomes, with reports describing both substantial clinical improvement and paradoxical psoriasis exacerbation [28,29,30].

## 7. Liraglutide

Among the studies evaluating GLP-1RAs in psoriasis, 11 publications specifically investigated the effects of liraglutide on psoriatic disease activity and associated metabolic parameters. These studies included case reports, prospective cohort studies, case-series analyses, and randomized controlled trials for a total of 87 patients treated with liraglutide-based regimens. Most studies focused on patients with concomitant obesity, insulin resistance, or T2DM, reflecting the well-established overlap between psoriasis and metabolic syndrome [14,15,19,20,25,33,34,35,36,37,38]. The majority of included patients presented with chronic plaque psoriasis, although severe and refractory forms were also reported, including pustular psoriasis, nail psoriasis, and extensive recalcitrant disease. Baseline psoriasis severity varied considerably between studies, with PASI scores ranging from mild disease (approximately PASI 4–5) to extremely severe forms exceeding PASI 30. Several patients had longstanding disease resistant to conventional therapies, including topical corticosteroids, vitamin D analogues, phototherapy, methotrexate, cyclosporine, fumaric acid esters, retinoids, and biologic agents such as etanercept and adalimumab. Liraglutide was administered subcutaneously in all studies, generally using gradual dose escalation protocols to improve tolerability. Most regimens started at 0.6 mg/day and were increased to 1.2 or 1.8 mg/day within 1–2 weeks. In obesity-oriented studies, escalation up to 3 mg/day was reported. Follow-up duration ranged from 6 weeks to 12 months, although most studies evaluated outcomes after approximately 12 weeks of treatment.

The results of our systematic review on liraglutide are set out in Table 6A,B.

Detailed study characteristics, metabolic outcomes and non-PASI clinical outcomes reported in liraglutide-treated patients with psoriasis are provided in Supplementary Table S5.

The effect of liraglutide on PASI is shown in Table 6C.

### 7.1. Clinical Outcomes

Overall, liraglutide therapy was associated with substantial clinical improvement in psoriasis severity in the majority of patients. The most robust evidence derived from the prospective cohort study by Xu et al., in which mean PASI decreased from 15.7 ± 11.8 to 2.2 ± 3.0 after 12 weeks of treatment [38]. In this cohort, 6 out of 7 patients achieved PASI50, while 5 patients achieved PASI75 [38]. Notably, one patient with severe pustular psoriasis and a baseline PASI of 31.3 demonstrated near-complete clinical resolution after 12 weeks of therapy [38]. Similarly, Lin et al. reported significant reductions in psoriasis severity in a randomized controlled trial involving psoriasis patients with T2DM, with mean PASI decreasing from 14.02 ± 10.67 to 2.40 ± 2.71 after 12 weeks of liraglutide treatment. PASI75 was achieved in 72.73% of treated patients [19]. Additional prospective studies and case reports confirmed these findings. Ahern et al. observed significant reductions in both PASI and DLQI scores after liraglutide therapy in obese patients with psoriasis and T2DM [35]. Case reports by Faurschou et al. and Reid et al. further demonstrated improvement in severe or treatment-refractory psoriasis following liraglutide initiation, including patients in whom immunosuppressive therapies were contraindicated because of malignancy [25,34]. In the case described by Reid et al., combination therapy with liraglutide and acitretin reduced PASI from 14.2 to 7.6 over 12 months in a patient with severe recalcitrant psoriasis and concomitant melanoma [25]. Buysschaert et al. reported clinical improvement associated with reductions in dermal γδ T-cell infiltration and IL-17 expression [14]. In their prospective case-series study, including patients treated with either liraglutide or exenatide, psoriasis improvement was associated with reduced dermal γδ T-cell infiltration and decreased IL-17 expression within psoriatic plaques [14]. Hogan et al. demonstrated increased circulating iNKT cells and reduced infiltration within psoriatic plaques following exenatide and liraglutide therapy, suggesting a role in the suppression of cutaneous inflammation [15]. Histopathological analyses additionally demonstrated reduced epidermal thickness, disappearance of Munro microabscesses, and decreased inflammatory infiltrates after treatment [19,38]. Several authors also reported reduced expression of pro-inflammatory cytokines, including IL-17, IL-23, and TNF-α, supporting a possible direct immunomodulatory role of liraglutide beyond its metabolic effects [14,19]. Beyond cutaneous improvement, liraglutide therapy consistently produced favourable metabolic effects. Significant reductions in body weight, BMI, waist circumference, HbA1c, fasting glucose, insulin resistance indices, and inflammatory markers were reported across multiple studies [14,19,35,37,38]. Weight loss ranged from approximately 3 to 10 kg during follow-up [19,25,34]. Improvements in DLQI paralleled PASI reductions, suggesting a meaningful impact on patient quality of life [19,35,38]. Biesenbach et al. demonstrated favourable effects of liraglutide on cardiovascular inflammatory markers in patients with type 2 diabetes [39]. Despite the predominantly favourable findings, one paradoxical adverse reaction was reported. Nowowiejska et al. described the first documented case of psoriasis exacerbation following liraglutide initiation in a 34-year-old woman with previously mild psoriasis [20]. Widespread erythematous-scaly lesions developed within 2 weeks of treatment initiation for insulin resistance, improving only after liraglutide discontinuation and cyclosporine therapy [20]. The authors hypothesized that cytokine imbalance and paradoxical immune activation, similar to phenomena observed during biologic therapy, could explain this event [20].

### 7.2. Safety Profile and Adverse Reactions

Overall, Liraglutide demonstrated a favourable safety profile across the included studies. Gastrointestinal adverse events, particularly nausea and vomiting during the dose-escalation phase, represented the most commonly reported side effects [14,15,19,34,35,38]. Less frequently reported adverse events included headache, constipation, and diarrhea [34]. Serious adverse events were rare, and no consistent safety concerns emerged across studies [14,15,19,20,25,33,34,35,36,37,38].

## 8. GLP-1 Receptor Agonists: Class-Wide Systemic Outcomes

Beyond drug-specific studies, several large real-world cohort studies evaluated the systemic and cardiovascular outcomes associated with GLP-1RAs as a pharmacological class in patients with psoriasis and associated metabolic comorbidities [40,41,42]. Unlike drug-specific reports primarily focused on cutaneous outcomes, class-wide studies mainly investigated cardiovascular events, mortality, and systemic inflammatory complications associated with GLP-1RA exposure. Most studies consisted of large retrospective population-based cohort analyses using real-world databases and propensity score-matched populations [40,41,42]. Follow-up duration ranged from approximately 2 [40] to 5 years [41] depending on study design. Overall, GLP-1RA therapy was consistently associated with favourable systemic outcomes. Olbrich et al. demonstrated significantly reduced all-cause mortality and lower risk of major adverse cardiovascular events (MACE) in psoriasis patients receiving GLP-1RAs compared with matched controls [40]. Similarly, Sontam et al. reported significantly lower risks of ischemic heart disease, stroke, peripheral artery disease, heart failure, and composite MACE across multiple follow-up periods in GLP-1RA-treated patients [41]. Hsiao et al. additionally demonstrated a significantly reduced incidence of psoriatic arthritis (PsA) in psoriasis patients with T2DM and/or obesity receiving GLP-1RAs compared with controls [42]. It should be noted that patients included in these population-based studies received heterogeneous concomitant psoriasis treatments, including topical therapies, conventional systemic agents, and biologic therapies, which may represent potential confounding factors when interpreting psoriasis-related outcomes [40,41,42].

## 9. Discussion

Current evidence suggests that GLP-1RAs may represent a promising therapeutic option for patients with psoriasis and concomitant metabolic comorbidities. Across the studies included in this review, treatment with GLP-1RAs was generally associated with clinical improvement in patients with obesity, T2DM and metabolic syndrome. In addition, the overall safety profile of this drug class appeared favourable. As summarized in Table 7, both systemic and cutaneous adverse events were uncommon, and reports of psoriasis improvement substantially outnumbered cases of disease worsening or de novo psoriasis occurring during treatment.

The mechanisms underlying the beneficial effects of GLP-1RAs on psoriasis remain incompletely understood. Both indirect metabolic effects and direct immunomodulatory actions are biologically plausible and are supported by varying degrees of experimental and clinical evidence. Improvements in psoriasis severity are frequently accompanied by weight loss, improved glycaemic control, and reductions in systemic inflammatory markers, making it difficult to determine the relative contribution of metabolic improvement versus direct immune modulation. Although some studies reported clinical improvement before substantial weight reduction occurred and translational data suggest effects on pathways involved in psoriasis pathogenesis, the currently available evidence remains insufficient to clearly disentangle these mechanisms. Indeed, molecular studies remain limited in number and have focused almost exclusively on liraglutide. Consequently, important knowledge gaps persist regarding the mechanisms through which GLP-1RAs may influence visceral adiposity, insulin resistance, systemic inflammation, and cutaneous disease activity in psoriasis. Potential mechanistic differences among individual agents also remain largely unexplored. Several differences emerged when individual drugs were considered separately. Semaglutide and liraglutide account for most of the currently available evidence in psoriatic patients. Among GLP-1RAs, semaglutide appears to exert particularly pronounced effects on body weight and metabolic parameters. Its long-acting pharmacologic profile may make it especially suitable for patients with psoriasis and severe metabolic dysfunction, including obesity, T2DM, and metabolic syndrome. Nevertheless, the biological mechanisms linking metabolic improvement to psoriasis outcomes remain poorly characterized. Few studies have investigated the molecular mediators potentially responsible for both the cutaneous and systemic effects of semaglutide. Further translational research, including serologic, histologic, molecular, genomic, and epigenetic investigations, may help us to clarify these mechanisms and improve our understanding of the relationship between metabolic dysfunction and psoriatic disease. Compared with semaglutide, somewhat greater mechanistic insight is available for liraglutide. Preclinical studies have suggested effects on iNKT cells, γδ T cells, and IL-17-related pathways [14,15,16]. Although these findings support a potential direct anti-inflammatory effect on psoriatic lesions, the available evidence remains limited and insufficient to define the precise molecular mechanisms involved. Additional preclinical and clinical studies are needed, particularly in patients without obesity or diabetes, in whom the relative contribution of weight-independent effects could be more clearly evaluated. Evidence supporting the use of exenatide and tirzepatide in psoriasis is considerably more limited. For exenatide, the available literature comprises only three treated patients and one reported case of a psoriasiform eruption [14,26,27]. Given the scarcity of data, no firm conclusions can be drawn regarding its efficacy or safety in psoriasis. The evidence base for tirzepatide is somewhat larger but remains substantially smaller than that available for semaglutide and liraglutide. Overall, tirzepatide appears to be effective and well tolerated in psoriatic patients. However, cases of psoriasis worsening or new-onset psoriasis have also been reported [28,30]. The case series by Gisondi et al. is of particular interest because it represents the only study specifically evaluating combination therapy with GLP-1RAs and biologic agents. In that study, combined treatment was associated with improvements in both psoriasis severity and metabolic outcomes [29]. Although the available evidence remains preliminary, combination therapy warrants further investigation. The potential complementary effects of biologic agents and GLP-1RAs on systemic and cutaneous inflammation may be clinically relevant. Future studies should evaluate combination therapy against biologic monotherapy and GLP-1RA monotherapy and explore whether concomitant GLP-1RA treatment could facilitate biologic dose reduction in selected patients. In this regard, an additional aspect that deserves consideration is the potential pharmacokinetic impact of the substantial weight loss achieved with newer GLP-1RAs. Obesity is known to influence the pharmacokinetics and clinical effectiveness of several biologic agents used in psoriasis, particularly fixed-dose therapies. Previous studies have shown that higher body weight, especially above 100 kg, is associated with lower rates of PASI90 and PASI100 responses, likely owing to increased drug clearance, larger volume of distribution, and the greater inflammatory burden associated with adiposity. Consequently, the 15–20% weight reductions commonly observed with semaglutide and tirzepatide may theoretically improve the effectiveness of concomitant biologic therapy by optimizing drug exposure. This potential pharmacokinetic and pharmacodynamic interaction has not yet been adequately investigated and may represent an additional mechanism through which GLP-1RAs contribute to improved psoriasis outcomes [43,44]. The available evidence does not allow identification of a superior GLP-1RA for psoriasis. No head-to-head comparative studies have been performed, and direct comparisons between agents are therefore not possible. Across the studies included in this review, complete skin clearance (PASI 0) was rarely achieved irrespective of the specific molecule used, as summarized in the PASI outcome tables. PASI 0 was reported only in the studies by Malavazos et al. evaluating semaglutide and by Gisondi et al. evaluating tirzepatide, the latter in combination with biologic therapy [12,29]. Although current psoriasis treatment goals increasingly emphasize complete or near-complete skin clearance, the available data suggest that GLP-1RA monotherapy is unlikely to achieve this outcome in most patients. Treatment duration should also be considered when interpreting efficacy outcomes. Several studies evaluating liraglutide reported PASI reductions of at least 50% within 12 weeks, whereas others observed only modest clinical improvement over the same period [19,33,37,38]. Notably, only four studies included treatment durations exceeding 24 weeks [12,18,22,25]. In two of these studies, final PASI values were below 5, raising the possibility that longer treatment exposure may be required to achieve optimal clinical benefit. Such findings are consistent with the hypothesis that sustained metabolic improvement may be necessary before its full impact on psoriasis becomes evident. Cases of GLP-1RA-induced psoriasis or psoriasiform eruptions remain uncommon and are currently supported only by isolated reports [20,26,28,30]. The clinical and pathogenic features of this phenomenon remain poorly characterized. Because cases have been described with multiple agents, a class-related mechanism cannot be excluded. An additional area of interest concerns patients with a history of malignancy. Liraglutide has been used successfully in patients in whom immunosuppressive therapies were contraindicated because of cancer [30,36]. Although several biologic agents have also demonstrated acceptable safety profiles in this setting [45], GLP-1RAs may represent a potential therapeutic option for selected patients requiring treatment of both psoriasis and metabolic disease. Hsiao et al. reported a lower incidence of psoriatic arthritis among obese or diabetic patients receiving GLP-1RAs compared with controls [42]. However, evidence regarding the effects of GLP-1RAs on established psoriatic arthritis remains extremely limited and is currently restricted to recent combination-therapy studies, highlighting the need for further dedicated investigations [32]. Finally, nearly all available studies have focused on plaque psoriasis. Evidence regarding other clinical variants remains extremely limited. Isolated reports involving liraglutide have described favorable outcomes in pustular and nail psoriasis, including marked improvement in a patient with pustular psoriasis after 12 weeks of treatment. However, the available data are insufficient to determine whether the efficacy of GLP-1RAs extends to non-plaque forms of psoriasis. Overall, the current literature suggests that GLP-1RAs may provide clinical benefit in selected patients with psoriasis, particularly those with concomitant obesity, T2DM, or other metabolic comorbidities. However, the available evidence is largely derived from case reports, small case series, and observational studies, with substantial methodological and clinical heterogeneity. Consequently, GLP-1RAs should not currently be considered primary treatments for psoriasis but rather potentially beneficial adjunctive agents in carefully selected patients. Larger prospective studies, mechanistic investigations, and comparative trials are needed to better define their dermatological efficacy, clarify the relative contribution of metabolic and immunological effects, and identify the patients most likely to benefit from this therapeutic approach.

## 10. Conclusions

Current evidence suggests that GLP-1 receptor agonists may provide clinical and metabolic benefits in selected patients with psoriasis, particularly those with obesity, T2DM, or other cardiometabolic comorbidities. Improvements in psoriasis severity have been reported across multiple studies, especially with semaglutide and liraglutide, although the available evidence remains limited and heterogeneous. While experimental and translational findings support potential immunomodulatory effects, the relative contribution of direct anti-inflammatory mechanisms versus improvements in metabolic status remains uncertain. At present, GLP-1RAs should not be considered primary therapies for psoriasis. Rather, they may represent valuable adjunctive therapeutic options in selected patients in whom metabolic and dermatological benefits can be pursued simultaneously. Given the predominance of case reports and small observational studies, the scarcity of randomized controlled trials, and the substantial heterogeneity of the available literature, firm conclusions regarding comparative efficacy among individual agents cannot yet be drawn. Larger prospective studies and adequately powered randomized trials are needed to clarify the role of GLP-1RAs in psoriasis management and to better define the patients most likely to benefit from these therapies.

## Figures and Tables

**Table 1:** Overview of currently marketed and emerging incretin-based therapies.

Agent	Target Receptor(s)	Development Status	Route of Administration
Exenatide	GLP-1 receptor agonist	Approved	Subcutaneous
Lixisenatide	GLP-1 receptor agonist	Approved	Subcutaneous
Liraglutide	GLP-1 receptor agonist	Approved	Subcutaneous
Dulaglutide	GLP-1 receptor agonist	Approved	Subcutaneous
Semaglutide	GLP-1 receptor agonist	Approved	Subcutaneous/Oral
Tirzepatide	Dual GIP/GLP-1 receptor agonist	Approved	Subcutaneous
Orforglipron	GLP-1 receptor agonist	Under development	Oral
Retatrutide	Triple GIP/GLP-1/glucagon receptor agonist	Under development	Subcutaneous
CagriSema	Semaglutide + cagrilintide combination	Under development	Subcutaneous

Abbreviations: GIP: glucose-dependent insulinotropic polypeptide; GLP-1: glucagon-like peptide-1. Note: Data adapted from Yao et al. and Latif [9,17].

**Table 2:** Pharmacological characteristics of GLP-1 receptor agonists included in this review.

Drug	Structural Characteristics	Molecular Weight (Da)	Route of Administration	Half-Life	Typical Dosing Regimen
Semaglutide	Modified human GLP-1 analogue with fatty-acid side chain prolonging albumin binding and resistance to DPP-4 degradation	4113.6	Subcutaneous/Oral	~168 h (7 days)	0.25–2.4 mg once weekly SC; 3–14 mg once daily oral
Liraglutide	Modified human GLP-1 analogue with C16 fatty-acid side chain	3751.2	Subcutaneous	~13 h	0.6–1.8 mg once daily
Exenatide	Synthetic exendin-4 analogue derived from *Heloderma suspectum* peptide	4186.6	Subcutaneous	~2.4 h (immediate-release formulation)	5–10 μg twice daily or 2 mg once weekly (extended-release)
Tirzepatide	Synthetic dual GIP/GLP-1 receptor agonist peptide	4813.5	Subcutaneous	~120 h (5 days)	2.5–15 mg once weekly

Abbreviations: DPP-4: dipeptidyl peptidase-4; GIP: glucose-dependent insulinotropic polypeptide; GLP-1: glucagon-like peptide-1; SC: subcutaneous. Note: Pharmacological characteristics adapted from Latif [9].

**Table 3:** (**A**) Characteristics, clinical outcomes, metabolic outcomes, and adverse events of semaglutide therapy in patients with psoriasis. (**B**) Effects of Semaglutide on PASI in the included studies. PASI values are reported as originally presented by the authors (single value, mean ± SD, median [IQR], or change from baseline).

(**A**)												
**Study ID**	**Authors (Year)**	**Type of Publication**	**Sex/Age**	**Psoriasis Type**	**Comorbidities**	**Previous Psoriasis Treatment**	**Drug’s Dose**	**Concomitant Therapies**	**Duration of Follow Up**	**Baseline PASI**	**Follow-Up PASI**	**Adverse Events**
1	G. Costanzo et al., 2021 [22]	CR	M; 73	plaque type	T2DM, chronic obstructive pulmonary disease	metformin 1000 mg 2/daily; Adalimumab	0.25 mg/week → 0.50 mg/week (week 4) → 1 mg/week (week 12)	Metformin	10 months	33.2	2.6	None
2	A. E. Malavazos et al., 2023 [12]	CR	F; 50	NR	T2DM, abdominal obesity	Ixekizumab, Secukinumab, Guselkumab	0.25 mg/week for 4 weeks → 0.50 mg/week for 16 weeks → 1 mg/week	None	10 months	12	0.2	None
3	B. Lin et al., 2025 [24]	CR	M; 49	plaque type	T2DM; MASLD	topical medication	0.25 mg/week → 0.5 mg/week (week 4) → 1 mg/week (week 8)	metformin 1000 mg daily, topical tretinoin cream	24 weeks	22	6.2 (near complete resolution)	Mild transient nausea during dose escalation (0.5 → 1 mg/week), lasting 8–10 days
4	Petkovic- Dabic et al., 2025 [23]	RCT	15 patients (12M, 3F); 58.6 ± 8.04 years;	Moderate-to-severe plaque psoriasis	T2DM	metformin	1.0 mg s.c. weekly	metformin	12 weeks	20.75 ± 9.72	10	2 patients excluded because of drug side effects [nausea, vomiting], and 1 due to exacerbation of the disease
5	J. Nicolau et al., 2026 [18]	PCS	43 patients (23F; 20M) mean age 46.7	Moderate-to-severe psoriasis (type NR)	obesity	Phototerapy [5]; Biological therapy [25];	0.25 mg weekly → monthly escalation up to a maximum of 2.4 mg weekly	Concomitant psoriasis therapies allowed if stable (NO recent modification of psoriasis treatment within the preceding 3 months or during follow-up)	6 months	10.6	5.5	Gastrointestinal adverse events
(**B**)												
**Authors (Year)**		**Type of Publication**			**Number of Patients**		**Baseline PASI (Mean/Median Value and SD or IQR or Value)**		**Endpoint PASI (Mean/Median Value and SD or IQR or Value)**		**Duration of Follow-Up**	
Costanzo et al., 2021 [22]		Case report			1		33.2		2.6		10 months	
Malavazos et al., 2023 [12]		Case report			1		12.0		0.2		10 months	
Lin et al., 2025 [24]		Case report			1		22.0		6.2		24 weeks	
Petković-Dabić et al., 2025 [23]		Randomised Controlled Trial			15		21 (19.8)		10		12 weeks	
Nicolau et al., 2026 [18]		Prospective Cohort Study			20		10.6 ± 7.1		5.5 ± 5.1		12 weeks	

Abbreviations: CR: case report; RCT, randomised controlled trial; PCS: prospective cohort study; PASI, Psoriasis Area and Severity Index; NR, not reported.

**Table 4:** (**A**) Characteristics, clinical outcomes, metabolic outcomes, and adverse events of exenatide therapy in patients with psoriasis. (**B**) Reported paradoxical psoriasis-related adverse reactions associated with exenatide therapy. (**C**) Effects of Exenatide on PASI in the included studies. PASI values are reported as originally presented by the authors (single value, mean ± SD, median [IQR], or change from baseline).

(**A**)														
**Study ID**	**Authors (Year)**	**Type of Publication**	**Sex/Age**	**Psoriasis Type**	**Comorbidities**	**Previous Psoriasis Treatment**	**Drug’s Dose**		**Concomitant Therapies**	**Duration of Follow-Up**	**Baseline PASI**	**Follow-Up PASI**		**Adverse Events**
1	M. Buysschaert et al., 2012 [27]	CR	M; 61	plaque type	T2DM	multiple steroid-based treatments	2 × 5 g/day		sulphonylureas and metformin	1 year	11	3–4 → worsened after treatment discontinuation (PASI > 10), and improved again after rechallenge (PASI 3.1 at 1 year)		NR
2	Buysschaert M. et al., 2014 [27]	Prospective cohort study	7 patients 5 males, 2 females Mean age: 56.8 years	plaque type	T2DM; Obesity/overweight (mean BMI ≈ 32 kg/m^2^)	NR	Exenatide 5 μg SC BID (1 patient) or liraglutide 0.6 mg/day for 1 week then usually 1.2 mg/day; increased to 1.8 mg/day if glycemic response insufficient		Metformin Sulfonylureas Insulin	18 weeks	Mean PASI: 12.0 ± 5.9	PASI 9.2 ± 6.4		Hypoglycaemic episodes (in insulin-treated patients)
(**B**)														
**Adverse/Paradoxical Cutaneous Effects**														
**Study ID**	**Authors (Year)**	**Type of Publication**	**Sex/Age**	**Psoriasis Type**	**Comorbidities**	**Previous Psoriasis Treatment**	**Drug’s Dose**	**Concomitant Therapies**	**Duration of Follow-Up**	**Baseline PASI**	**Baseline Non-PASI Outcomes**	**Follow-Up PASI**	**Follow-Up Non-PASI Outcomes**	**Adverse Events**
3	E. Bostan et al., 2022 [26]	CR	F; 56	New-onset psoriasiform dermatitis 4 days after the administration of exenatide	T2DM	None	2 × 5 μg/day	Metformin	NR	NR	NR	NR	NR	NR
(**C**)														
**Authors (Year)**		**Type of Publication**				**Number of Patients**			**Baseline PASI (Mean/Median Value and SD or IQR or Value)**			**Endpoint PASI (Mean/Median Value and SD or IQR or Value)**		
M. Buysschaert et al., 2012 [27]		Case report				1			11			3.1		

Abbreviations: BID, bis in die/twice daily; BMI, body mass index; CR, complete remission; M, male; NR, not reported; PASI, Psoriasis Area and Severity Index; SC, subcutaneous; T2DM, type 2 diabetes mellitus; BMI, body mass index; CR, case report; DLQI, Dermatology Life Quality Index; F, female; HbA1c, glycated haemoglobin; NR, not reported; PASI, Psoriasis Area and Severity Index; T2DM, type 2 diabetes mellitus.

**Table 5:** (**A**) Characteristics, clinical outcomes, metabolic outcomes, and adverse events of tirzepatide therapy in patients with psoriasis. (**B**) Reported paradoxical psoriasis-related adverse reactions associated with tirzepatide therapy. (**C**) Effects of Tirzepatide on PASI in the included studies. PASI values are reported as originally presented by the authors (single value, mean ± SD, median [IQR], or change from baseline).

(**A**)														
**Study ID**	**Authors (Year)**	**Type of Publication**	**Sex/Age**	**Psoriasis Type**	**Comorbidities**		**Previous Psoriasis Treatment**	**Drug’s Dose**	**Concomitant Therapies**	**Duration of Follow-Up**	**Baseline PASI**	**Follow-Up PASI**		**Adverse Events**
1	Gisondi et al., 2025 [29]	CS	10 patients (6M, 4F); 53.3 years	moderate- to- severe plaque psoriasis	obesity		biological treatment for at least 12 months	2.5 mg/week for 1 month → 5 mg/week for 5 months	Biological therapy was maintained stable (Secukinumab, adalimumab, ustekinumab, guselkumab, tildrakizumab)	6 months	6.4 ± 0.68	0.3 ± 0.75 (95% reduction)		Mild nausea (*n* = 3); intermittent diarrhoea (*n* = 1)
2	Lebwohl et al., 2026 [31]	Randomized open-label phase 3b clinical trial	123 F/151 M; mean age 45.6 ± 12.7 years	Moderate-to-severe plaque psoriasis	Overweight/obesity; hypertension (44.5%), dyslipidaemia (20.1%), T2DM (10.9%), cardiovascular disease (4.7%), PsA (12.4%)		34.3% previously treated with advanced therapies	Ixekizumab + tirzepatide (2.5–15 mg weekly SC)	Ixekizumab	36 weeks	Mean PASI 19.7 ± 8.1	PASI100 40.6%; PASI90 75.6%; PASI75 91.5%; mean PASI change −18.1; mean PASI reduction −92.2%		Treatment-emergent AEs in 71.0%; nausea (24.6%), diarrhoea (14.5%), constipation (13.0%), vomiting (8.7%); serious AEs 3.6%; discontinuation due to AEs 3.6%; no new safety concerns
(**B**)														
**Adverse/Paradoxical Cutaneous Effects**														
**Study ID**	**Authors (Year)**	**Type of Publication**	**Sex/Age**	**Psoriasis Type**	**Comorbidities**	**Previous Psoriasis Treatment**	**Drug’s Dose**	**Concomitant Therapies**	**Duration of Follow-Up**	**Baseline PASI**	**Baseline Non-PASI Outcomes**	**Follow-Up PASI**	**Follow-Up Non-PASI Outcomes**	**Adverse Events**
3	Cook & Selby 2025 [30]	CR	F; 59	Guttate/inverse psoriasiform eruption	Obesity	None	Tirzepatide started 7 months prior and dose increased 3 months before eruption	Clobetasol 0.05% ointment, fluocinonide 0.05% scalp solution, ixekizumab 80 mg/mL, roflumilast 0.3% cream → risankizumab-rzaa 150 mg/mL at 6 weeks fu, roflumilast	~5 months	NR (~40% BSA involvement)	NR	NR	Psoriasis reduced from 40% to 4% BSA, then complete resolution	Tirzepatide-induced psoriasis
4	R. Neptune et al., 2026 [28]	CR	F; 37	Plaque psoriasis flare (3-month history of a new, progressively pruritic generalized eruption; started 4 weeks after initiation of tirzepatide for weight loss)	Hypertension, rosacea	Topical halobetasol 0.05% ointment had previously been pre scribed an as-needed basis but was not in active use	NR	Lisinopril/HCTZ, topical metronidazole. After the eruption: methotrexate → adalimumab → ixekizumab	~6 months	NR	Stable psoriasis in remission > 6 months before tirzepatide	NR	NR	Psoriatic flare
(**C**)														
**Authors (Year)**		**Type of Publication**				**Number of Patients**			**Baseline PASI (Mean/Median Value and SD or IQR or Value)**			**Endpoint PASI (Mean/Median Value and SD or IQR or Value)**		
Gisondi et al., 2025 [29]		Case series				10			6.4 ± 0.68			0.3 ± 0.75		
Lebwohl et al., 2026 [31]		Randomized open-label phase 3b clinical trial				274			19.7 ± 8.1			PASI100 40.6%; PASI90 75.6%; PASI75 91.5%;		

Abbreviations: BMI, Body Mass Index; DLQI, Dermatology Life Quality Index; PASI, Psoriasis Area and Severity Index. PsA: psoriatic arthritis; SC: subcutaneous; T2DM: type 2 diabetes mellitus; AEs: adverse events. Note: In the study by Lebwohl et al. [31], Tirzepatide was administered as add-on therapy to Ixekizumab. Therefore, psoriasis outcomes reflect the effect of combination therapy (ixekizumab plus tirzepatide) rather than tirzepatide monotherapy. Abbreviations: CR: case report; BMI, Body Mass Index; BSA, body surface area; DLQI, Dermatology Life Quality Index; NR, not reported; PASI, Psoriasis Area and Severity Index.

**Table 6:** (**A**) Characteristics, clinical outcomes, metabolic outcomes, and adverse events of liraglutide therapy in patients with psoriasis. (**B**) Reported paradoxical psoriasis-related adverse reactions associated with liraglutide therapy. (**C**) Effects of Liraglutide on PASI in the included studies. PASI values are reported as originally presented by the authors (single value, mean ± SD, median [IQR], or change from baseline).

(**A**)														
**Study ID**	**Authors (Year)**	**Type of Publication**	**Sex/Age**	**Psoriasis Type**	**Comorbidities**	**Previous Psoriasis Treatment**			**Drug’s Dose**	**Concomitant Therapies**	**Duration of Follow Up**	**Baseline PASI**	**Follow-Up PASI**	**Adverse Events**
1	Faurschou A. et al., 2014 [34]	CR	M; 59	plaque type	T2DM; hypertension; hypercolesterolemia; myocardial infarction	topical steroids; nbUVB			0.6 mg/day → week 0 1.2 mg/day → after 1 week 1.8 mg/day → after 5 weeks	metformin 1000 mg BID, insulin	12 weeks	NR	NR	Nausea; Headache (resolved within 1–2 months); Intermittent constipation and diarrhoea
2	Hogan A.E. et al., 2011 [15]	CS	Male, 48 years Male, 49 years	plaque type	T2DM (all patients); Obesity (BMI up to 48 kg/m^2^); Hypertension (both patients) Dyslipidaemia (1 patient) Osteoarthritis (1 patient)	No psoriasis treatment in previous 6 months			NR	Metformin (all), Aspirin Antihypertensives (ACE inhibitors, calcium channel blockers, diuretics) Statins	6 weeks	Patient 1: 13.2 Patient 2: 4.8	Patient 1: 10.8 (6 weeks) Patient 2: 3.8 (6 weeks)	None
3	Ahern T. et al., 2013 [35]	Prospective cohort study	7; 5 males, 2 females; Age: Median 48 years (IQR 40–58)	plaque type	T2DM (all patients) Obesity (majority severely obese, BMI > 40 kg/m^2^)	No prior systemic therapy No active topical therapy during study			0.6 mg/day for 2 weeks --> 1.2 mg/day (maintenance dose)	NR	10 weeks	Median PASI: 4.8 (IQR 2.6–11.4)	Median PASI: 3.0 (IQR 1.9–7.9)	No serious adverse events reported
4	Reid C.T et al., 2013 [25]	CR	M; 54	plaque type	IFG; Obesity (BMI > 40); malignant melanoma	Etanercept; efalizumab. nbUVB, psoralen–ultraviolet A, closporin, methotrexate, acitretin; fumaric acid esters; Adalimumab			0 6 mg daily sucutaneously for 1 week, gradually increased over 6 months to 3 mg daily		12 months	14.2	7.6	No adverse event reported
5	Buysschaert M. et al., 2014 [27]	Prospective case series study	7 5 males, 2 females Mean age: 56.8 years	plaque type	T2DM; Obesity/overweight (mean BMI ≈ 32 kg/m^2^)	NR			Exenatide 5 μg SC BID (1 patient) or liraglutide 0.6 mg/day for 1 week then usually 1.2 mg/day; increased to 1.8 mg/day if glycemic response insufficient	Metformin Sulfonylureas Insulin	18 weeks	Mean PASI: 12.0 ± 5.9	PASI 9.2 ± 6.4	Hypoglycaemic episodes (in insulin-treated patients)
6	Faurschou A. et al., 2015 [36]	RCT	Total: 20 Liraglutide group: 11 Placebo group: 9; Sex: 15 males, 5 females; Age: Placebo: 48 ± 12 years Liraglutide: 54 ± 14 years	plaque type	Obesity/overweight (BMI > 25; mean ~35–37 kg/m^2^)	NR			Liraglutide: 0.6 mg/day (week 1) → 1.2 mg/day (week 2) → 1.8 mg/day (remaining 6 weeks)	Topical corticosteroids/vitamin D analogues (some patients) 1 patient on adalimumab	8 weeks	Placebo: 11.6 ± 5.5 Liraglutide: 14.5 ± 7.2	Liraglutide: −2.6 ± 2.1 Placebo: −1.3 ± 2.4	Nausea (45%) Loss of appetite (18%) Constipation (9%)
7	Xu X. et al., 2019 [38]	Prospective cohort study	Total: 7 patients 6 males, 1 female Mean age: 60 ± 8 years	plaque type	T2DM	None			Start: 0.6 mg/day After 1 week → 1.2 mg/day Then → 1.8 mg/day	None	3 months	Mean: 15.7 ± 11.8 Range: 1.5–31.3	Mean: 2.2	Nausea/vomiting: 57% (early phase)
8	Lin L. et al., 2022 [19]	RCT	Total: 24 Control: 13 Liraglutide: 11 Sex: Control: 11 M/2 F Treatment: 10 M/1 F Age: Control: 55.23 ± 7.84 years Treatment: 56.73 ± 8.27 years	plaque type	T2DM	NR			Starting dose: 0.6 mg/day Escalation: +0.6 mg/week Max: 1.8 mg/day	Control group: Acitretin 30–50 mg/day Calcipotriol topical Treatment group: Liraglutide + oral hypoglycemics: Metformin ± glimepiride OR sitagliptin	3 months	Control: 13.57 ± 5.49 Treatment: 14.02 ± 10.67	Control: 13.57 → 7.42 Liraglutide: 14.02 → 2.40	GI symptoms (nausea/vomiting): 54.5%
9	Ramakrishnan et al., 2020 [33]	CR	M; 42	plaque type	T2DM; hypertension	topical therapy with an emollient and Mometasone and 3% salicylic acid on inflamed lesions			0.6 mg for 1 week → 1.2 mg daily	Metformin 1 g twice daily, Glimepride 2 mg twice daily; Telmisartan 40 mg once daily	4 weeks	10.2	4.7 (PASI50 response)	NR
10	Nicolau J. et al., 2023 [37]	Prospective cohort study	20;55% female (11/20), 45% male Mean age: 45.4 ± 9.7 years	plaque type; 30% psoriatic arthritis	Obesity (all patients) Psoriatic arthritis (30%) Depressive disorder (30%) Hypertension (35%) Dyslipidemia (20%) Obstructive sleep apnea (10%)	All patients actively treated: Biologics (16/20) Phototherapy (4/20)			0.6 mg/day (week 1) Weekly escalation → 3 mg/day target dose	Biologic therapy (majority) Phototherapy Lifestyle intervention: Hypocaloric diet (−500 kcal/day) ≥150 min/week exercise	3 months	10 ± 8.4	5.1 ± 6	GI side effects
(**B**)														
**Study ID**	**Authors (Year)**	**Type of Publication**	**Sex/Age**	**Psoriasis Type**	**Comorbidities**	**Previous Psoriasis Treatment**	**Drug’s Dose**	**Concomitant Therapies**	**Duration of Follow Up**	**Baseline PASI**	**Baseline Non-PASI Outcomes**	**Follow-Up PASI**	**Follow-Up Non-PASI Outcomes**	**Adverse Events**
11	Nowowiejska J. et al., 2023 [20]	CR	F; 34	history of mild psoriasis → after 2 weeks of liraglutide new psoriatic lesions appeared (numerous erythematous-scaly lesions on face and scalp and in the intertriginous areas)	Insulin resistance	None	NR	Cyclosporin A	4 months	NR	NR	NR	NR	Severe exacerbation of psoriasis
(**C**)														
**Authors (Year)**		**Type of Publication**				**Number of Patients**			**Baseline PASI (Mean/Median Value and SD or IQR or Value)**			**Endpoint PASI (Mean/Median Value and SD or IQR or Value)**		
Hogan A.E. et al., 2011 [15]		Case series				2			Patient 1: 13.2 Patient 2: 4.8			Patient 1: 10.8; Patient 2: 3.8		
Ahern T. et al., 2013 [35]		Prospective cohort study				7			4.8 (I2.6–11.4)			3.0 (I1.9–7.9)		
Reid C.T et al., 2013 [25]		Case report				1			14.2			7.6		
Faurschou A. et al., 2015 [36]		Randomized Controlled Trial				Total: 20 Liraglutide group: 11 Placebo group: 9			14.5 ± 7.2			−2.6 ± 2.1		
Xu X. et al., 2019 [38]		Prospective cohort study				7			15.7 ± 11.8			2.2 ± 3.0		
Lin L. et al., 2022 [19]		Randomized Controlled Trial				Total: 24 Control group: 13 Liraglutide group: 11			14.02 ± 10.67			14.02 → 2.40		
Ramakrishnan et al., 2020 [33]		Case report				1			10.2			4.7		
Nicolau J. et al., 2023 [37]		Prospective cohort study				20			10 ± 8.4			5.1 ± 6		

Abbreviations: ACE, angiotensin-converting enzyme; AE, adverse events; BID, twice daily; CR, case report; CS, case series; RCT, randomized controlled trial; nbUVB, narrowband ultraviolet B; NR, not reported; PASI, Psoriasis Area and Severity Index; PUVA, psoralen plus ultraviolet A; T2DM, type 2 diabetes mellitus; Abbreviations: BMI, Body Mass Index; DLQI, Dermatology Life Quality Index; NR, not reported; PASI, Psoriasis Area and Severity Index; NbUVB, narrowband ultraviolet B; BMI: body mass index.

**Table 7:** Drug-specific safety findings across included studies.

Drug	Reported Adverse Events	Psoriasis Worsening/Paradoxical Event	Discontinuation/Exclusion
Semaglutide	Gastrointestinal symptoms, mainly nausea and vomiting; constipation	One withdrawal for psoriasis exacerbation in the randomized trial; no paradoxical event clearly attributed across the other reports	Two exclusions for nausea/vomiting; one exclusion for psoriasis exacerbation
Exenatide	Hypoglycaemic episodes in insulin-treated patients	New onset psoriasiform dermatitis reported in one case	Psoriasiform dermatitis improved after drug withdrawal
Tirzepatide	Mild nausea and intermittent diarrhoea	Psoriasiform eruption or psoriasis flare reported in two cases	Drug withdrawal reported in paradoxical cutaneous cases
Liraglutide	Gastrointestinal symptoms, headache, loss of appetite; constipation, hypoglycaemia in insulin-treated patients	Severe psoriasis exacerbation reported in one case	No serious adverse events reported in selected studies; discontinuation not consistently reported

## Data Availability

No new data were created or analyzed in this study. All data supporting the findings of this review are available within the cited articles and the present manuscript.

## Supplementary Materials

The following supporting information can be downloaded at: https://www.mdpi.com/article/10.3390/jcm15135126/s1, Supplementary Table S1: Quality assessment and risk-of-bias evaluation of the included studies. Supplementary Table S2: Detailed study characteristics, non-PASI clinical outcomes and, metabolic outcomes reported in semaglutide-treated patients with psoriasis. Supplementary Table S3: Detailed study characteristics, non-PASI clinical outcomes and, metabolic outcomes reported in exenatide-treated patients with psoriasis. Supplementary Table S4: Detailed study characteristics, non-PASI clinical outcomes and, metabolic outcomes reported in tirzepatide-treated patients with psoriasis. Supplementary Table S5: Detailed study characteristics, non-PASI clinical outcomes and, metabolic outcomes reported in liraglutide-treated patients with psoriasis.
