# Supplementary material for: Effects of GLP-1 Receptor Agonists on Psoriasis: An “Agent-Specific” Systematic Review of the Literature
Source: J Clin Med. 2026 Jul 1;15(13):5126. doi: 10.3390/jcm15135126 (PMC13362826; doi:10.3390/jcm15135126)
Supplement: Supplementary file 1 [file jcm-15-05126-s001.zip › jcm-4385425-supplementary.pdf]

Supplementary Table S1. Quality assessment and risk-of-bias evaluation of the included studies.

Study ID	Type of publication	Assessment tool	Score / judgement	Overall quality/ risk of bias
Costanzo et al., 2021	Case report	JBI Case Report Checklist	8/8	High quality / Low risk of bias
Malavazos et al., 2023	Case report	JBI Case Report Checklist	8/8	High quality / Low risk of bias
Lin et al., 2025	Case report	JBI Case Report Checklist	8/8	High quality / Low risk of bias
Petković-Dabić et al., 2025	Open-label Randomized Clinical Trial	RoB 2	Some concerns	Moderate quality / Moderate risk of bias
Nicolau et al., 2025	Prospective cohort study	NOS	6/9	Moderate quality / moderate risk of bias
Faurschou et al., 2011	Case report	JBI Case Report Checklist	8/8	High quality / Low risk of bias
Ramakrishnan et al., 2020	Case report	JBI Case Report Checklist	7/8	High quality / Low risk of bias
Nowowiejska et al.	Case report	JBI Case Report Checklist	7/8	High quality / Low risk of bias
Reid et al., 2013	Case report	JBI Case Report Checklist	7/8	High quality / Low risk of bias
Hogan et al., 2011	Case series	JBI Case Series Checklist	8/10	High quality / Low risk of bias
Buysschaert et al., 2014	Prospective case series	JBI Case Series Checklist	9/10	High quality / Low risk of bias
Ahern et al., 2012	Prospective cohort study	NOS	6/9	Moderate quality / Moderate risk of bias
Xu et al., 2019	Prospective cohort study	NOS	6/9	Moderate quality / Moderate risk of bias
Nicolau et al., 2023	Prospective cohort study	NOS	6/9	Moderate quality / Moderate risk of bias
Faurschou et al., 2014	Randomized placebo-controlled trial	RoB 2	Low risk	High quality / Low risk of bias
Lin et al., 2020	Randomized controlled trial	RoB 2	Some concerns	Moderate quality / Moderate risk of bias
Buysschaert et al., 2012	Case report	JBI Case Report Checklist	8/8	High quality / Low risk of bias
Bostan et al., 2022	Case report	JBI Case Report Checklist	8/8	High quality / Low risk of bias
Neptune Rosa et al., 2026	Case report	JBI Case Report Checklist	8/8	High quality / Low risk of bias
Cook & Selby, 2025	Case report	JBI Case Report Checklist	8/8	High quality / Low risk of bias
Gisondi et al., 2025	Prospective case series	JBI Case Series Checklist	9/10	High quality / Low risk of bias

Legend: JBI = Joanna Briggs Institute Critical Appraisal Checklists for Case Reports and Case Series. High quality was defined as fulfilment of ≥75% of appraisal criteria;moderate quality/moderate risk of bias as fulfilment of 50–74% of appraisal criteria; low quality/high risk of bias as fulfilment of <50% of appraisal criteria.NOS, Newcastle–Ottawa Scale. High quality was defined as 7–9 stars, moderate quality as 5–6 stars, and low quality as <5 stars; RoB 2 = revised Cochrane Risk of Bias tool for randomized trials. Studies judged as "low risk" were considered high quality, those with "some concerns" were considered moderate quality, and those with "high risk" were considered low quality.

Supplementary Table S2: Detailed study characteristics, non-PASI clinical outcomes and, metabolic outcomes reported in semaglutide-treated patients with psoriasis.

Study ID	Authors (Year)	Type of publication	Duration of Follow up	Baseline Non-PASI Outcomes	Follow-up non-PASI Outcomes
1	G. Costanzo et al. 2021	CR	10 months	DLQI 26; type 3 obesity (BMI ≥40)	HbA1c: 5.4%; BMI:38.3; DLQI: 0
2	A. E. Malavazos et al. 2023	CR	10 months	DAPSA 31; DLQI 20; BMI 30.4 kg/m2; HBA1c 6.5%, CRP 4.3 mg/dL; TC 222 mg/dL, CT-EAT -80 HU; CT-PCAT LAD −65 HU; CT-PCAT Cx −77 HU; CT-PCAT RCA −68 HU	DAPSA 4; DLQI 1; BMI 22.6 kg/m2 ; HBA1c 5.1%; CRP <0.5 mg/dL;TC 196 mg/dl; CT-EAT -94 HU; CT-PCAT LAD −70 HU; CT-PCAT Cx artery -82HU; CT-PCAT RCA -72HU
3	B. Lin et al. 2025	CR	24 weeks	DLQ1 27; HbA1c 7.8%; BMI 26.18 kg/m2	HbA1c: 6.5%; BMI: 24.0 kg/m²; DLQI: 8; liver enzymes normalized
4	J. P. Dabic et al. 2025	RCT	12 weeks	BMI 35.04±5.9 kg/m2;; DLQI 13±6.5, HbA1c 7.3; Glucose 7mmol/L; TC 5.2±1.1 mmol/L; LDL3.6±1.1 mmol/l; CRP 3,8 mg/L; IL6 3.5 pg/mL	BMI 30.7±3.8 kg/m2; 13(46%) patients achieved PASI90, 1 (8%) PASI100; DLQI 4; TC 4.6±1.2 mmol/L; LDL 2.8 ± 0,9 mmol/L; HbA1c 6,1%; Glucose 7,3mmol/; CRP 1,9 mg/L; IL6 2.8 pg/mL
5	J. Nicolau et al. 2025	PCS	6 months	DLQI 11.7, VAS 5.2; BDI 15, BMI 38.6 kg/m2; PPF 1.8 cm, SAT 4 cm, HbA1c 5.6%; TC181.3mg dL–1; *hs-CRP* 3.8 mg L–1; *Hcy* 12.7 μmol L–1	DLQI 5; VAS 2.3; BDI 9.4; BMI 34.2; kg/m2; PPF 1.2 cm; SAT 2.7 cm; HbA1c 5.4%; TC 176. 2 mg dL–1, *hs-CRP* 1.9 mg L–1; *Hcy* 11.5 μmol L–1

Abbreviations: CR: case report; RCT, randomised controlled trial;PCS: Prospective Cohort Study ;PASI, Psoriasis Area and Severity Index; DAPSA = Disease Activity in Psoriatic Arthritis Score; DLQI, Dermatology Life Quality Index; NR, not reported; BMI, Body Mass Index; CRP, C-reactive protein; TC: total cholesterol; EAT: epicardial adipose tissue attenuation (HU); PCAT : pericoronary adipose tissue attenuation; LAD: left anterior descending artery; Cx:circumflex artery;RCA: right coronary artery; PPF: Preperitoneal Fat; SAT = Subcutaneous Adipose Tissue · hs-CRP = High-sensitivity C-reactive Protein; Hcy = Homocysteine

Supplementary Table S3: Detailed study characteristics, non-PASI clinical outcomes and, metabolic outcomes reported in exenatide-treated patients with psoriasis.

Study ID	Authors (Year)	Type of publication	Duration of Follow-up	Baseline Non-PASI Outcomes	Follow-up PASI
1	M. Buysschaert et al. 2012	CR	1 year	HbA1c 7.6%; BMI: 25.5 kg/m2; CRP 0.22 mg/dL	3-4--> worsened after treatment discontinuation (PASI >10), and improved again after rechallenge (PASI 3.1 at 1 year)
2	Buysschaert M. et al; 2014	Prospective cohort study	18 weeks	BMI: 32.0 ± 10.1 kg/m² HbA1c: 7.5 ± 1.2% Immunological: ↑ dermal γδ T cells ↑ IL-17 expression (vs control skin)	PASI 9.2 ± 6.4

Abbreviations: BID, bis in die/twice daily; BMI, body mass index; CR, complete remission; CRP, C-reactive protein; DLQI, Dermatology Life Quality Index; HbA1c, glycated haemoglobin; IL, interleukin; IL-17, interleukin-17; M, male; NR, not reported; PASI, Psoriasis Area and Severity Index; SC, subcutaneous; T2DM, type 2 diabetes mellitus

Supplementary Table S4: Detailed study characteristics, non-PASI clinical outcomes and, metabolic outcomes reported in tirzepatide-treated patients with psoriasis.

Study ID	Authors (Year)	Type of publication	Duration of Follow-up	Baseline Non-PASI Outcomes	Follow-up non-PASI Outcomes
1	Gisondi et al. 2025	CS	6 months	BMI ≥30 kg/m2 (weight 107.2 ± 7 kg); DLQI 6.9 ± 0.7 ; WC 116 ±7 cm; LDL 104 ±11 mg/dL; TG 181 ±23 mg/dL; Gly 96 ± 6mg/dL	DLQI 1.3 ± 0.6, (81% of ↓); weight 94.1 ± 5.9 kg (↓); WC 104 cm ±6 (↓); LD L 98 ±10 mg/dL (↓); TG 146 ±19mg/dL (↓) ; Gly 94 ± 6 mg/dL (↓)
2	Lebwohl et al. 2026	Randomized open-label phase 3b clinical trial	36 weeks	DLQI 11.1 ± 6.7; BMI 39.2 ± 9.1 kg/m²; body weight 113.8 ± 28.5 kg; HbA1c 5.8 ± 0.8%; triglycerides 169.9 ± 104.0 mg/dL	DLQI 0/1 in 71.7%; sPGA 0/1 in 76.5%; ≥4-point itch NRS improvement 81.8%; body weight −14.6%; BMI −5.71 kg/m²; SBP −9.9 mmHg; DBP −3.6 mmHg; total cholesterol −10.0 mg/dL; triglycerides −32.2 mg/dL; HbA1c −0.5%

Abbreviations: BMI, Body Mass Index; DLQI, Dermatology Life Quality Index; PASI, Psoriasis Area and Severity Index. LDL, low-density lipoprotein cholesterol; TG, triglycerides; Gly, glycaemia ; WC: waist circumference; DBP: diastolic blood pressure; HbA1c: glycated haemoglobin; NRS: Numeric Rating Scale; PsA: psoriatic arthritis; SBP: systolic blood pressure; SC: subcutaneous; sPGA: static Physician Global Assessment; T2DM: type 2 diabetes mellitus.

Supplementary Table S5: Detailed study characteristics, non-PASI clinical outcomes and, metabolic outcomes reported in liraglutide-treated patients with psoriasis.

Study ID	Authors (Year)	Type of publication	Duration of Follow up	Baseline non-PASI Outcomes	Follow-up non-PASI Outcomes
1	Faurschou A. et al., 2011	CR	12 weeks	HbA1c: 8.9% (74 mmol/mol)<br>BMI: 29.3 kg/m²<br>Weight: 91.8 kg	HbA1c: 5.9% (41 mmol/mol)<br>Weight: 84.0 kg
2	Hogan A.E. et al; 2011	CS	6 weeks	BMI: 48.0 and 43.0<br>Weight:<br>159.1 kg / 137.8 kg<br>HbA1c:<br>~5.7–5.9%	Weight: ↓ (159.1 → 154.0 kg; 137.8 → 131.6 kg)<br>BMI:<br>↓ (48 → 46.5; 43 → 41.1)<br>↑ circulating iNKT cells<br>↓ iNKT cells in psoriatic plaques
3	Ahern T. et al., 2012	Prospective cohort study	10 weeks	DLQI: median 6.0 (IQR 3.5–8.9)<br>BMI: median 48.2 kg/m²<br>Weight: median 137.8 kg (IQR 120–178)<br>FG: 6.1 mmol/L (median)	DLQI: median 2.0 (IQR 1–6.1)<br>Weight: median 130.1 kg (~5% reduction)<br>FG: 5.8 mmol/L<br>iNKT cells: increased (0.13% → 0.40%)<br>TNF-α–producing monocytes: ↓ ~53% (NS)
4	Reid C.T et al; 2013	CR	12 months	DLQI: 25	DLQI: 12; weight reduced of 10 kg
5	Buysschaert M. et al; 2014	Prospective case series study	18 weeks	BMI: 32.0 ± 10.1 kg/m²<br>HbA1c: 7.5 ± 1.2%<br>Immunological:<br>↑ dermal γδ T cells<br>↑ IL-17 expression (vs control skin)	BMI: ↓ to 30.6 ± 9.1 kg/m²<br>HbA1c: ↓ to 6.5 ± 0.8%<br>Histology:<br>↓ epidermal thickness (trend)<br>Immunological:<br>γδ T cells: ↓ (6.7% → 2.7%, p=0.05)<br>IL-17: reduced
6	Faurschou A. et al., 2014	RCT	8 weeks	DLQI change :<br>Placebo: 7.9 ± 4.5<br>Liraglutide: 8.7 ± 6.1<br>BMI:<br>Placebo: 35 ± 11.5<br>Liraglutide: 37 ± 8.2<br>Weight:<br>Placebo: 102 kg<br>Liraglutide: 116 kg<br>HbA1c: ~5.4–5.7%<br>hsCRP:<br>Placebo: 3.8 mg/L<br>Liraglutide: 5.4 mg/L	DLQI change:<br>Liraglutide: −2.5 ± 4.4<br>Placebo: −3.7 ± 4.8 → NS<br>Weight:<br>Liraglutide: −4.7 ± 2.5 kg<br>Placebo: −1.6 ± 2.7 kg (significant)<br>Cholesterol: ↓ significantly with liraglutide<br>HbA1c: no significant change<br>Fasting glucose: slight decrease (liraglutide)<br>hsCRP: no change
7	Xu X. et al; 2019	Prospective cohort study	3 months	DLQI 21.8 ± 6; BMI 23 ± 4; WC 87 ± 9 cm; HbA1c 8.1 ± 2.3%; FG 6.2 mmol/L; C-peptide 1.4 ± 0.7 ng/mL; HOMA-IR 2.8 ± 0.8; LDL 2.6 ± 0.5 mmol/L; CRP 28 ± 43 mg/L	DLQI 4.1 (p=0.001); BMI 21 (p<0.01); WC 83 cm (p<0.05); HbA1c 6.4% (p=0.04); FG ↓; C-peptide 1.9 (p=0.006); HOMA-IR 1.6 (p=0.03); LDL ↓; CRP ↓; ET ↓; resolution of Munro abscesses and neutrophilic infiltrate
8	Lin L. et al., 2020	RCT	3 months	DLQI:<br>Control: 18.23 ± 5.17<br>Treatment: 22.00 ± 5.85<br>BMI:<br>~23.7 kg/m²<br>Weight:<br>~66 kg<br>HbA1c:<br>Control: 7.30 ± 1.88%<br>Treatment: 7.80 ± 2.55%	DLQI:<br>Control: 9.69<br>Liraglutide:3.82<br>Weight: ↓−4.82 kg<br>BMI: ↓<br>Waist circumference: ↓<br>HbA1c: ↓<br>HOMA-IR: ↓<br>C-peptide: ↓<br>TC, LDL, TG: ↓<br>↓ epidermal thickness<br>↓ inflammatory infiltrate<br>↓ IL-17, IL-23, TNF-α expression
9	Ramakrishnan et al. 2020	CR	4 weeks	BMI 32.5 kg/m2; HbA1c 8.4%; FPG 132mg/dl ; PPPG 228 mg/ dl	FPG 102 mg/dl; PPPG 142 mg/dl; weight loss of 3 kg
10	Nicolau J. et al; 2023	Prospective cohort study	3 months	BMI: 38.9 ± 5.8 kg/m²; DLQI: 12.7 ± 7;<br>VAS (pain): 4.1 ± 2<br>CRP: 4.5 ± 2.4 mg/L;<br>Hcy: 13.3 ± 3.6 µmol/L<br>Ferritin: 185.4 ± 142.2 ng/mL<br>Cortisol: 12 ± 3.1 µg/dL<br>HbA1c: 5.5 ± 0.3%	BMI: 36.4 ± 5.6 kg/m² ;DLQI: 6.4 ± 5.6<br>VAS: 2.3 ± 0.92; CRP: ↓ to 3 mg/L<br>Hcy: ↓ to 11.9; Ferritin: ↓ to 97.4<br>Cortisol: ↓ slightly; HbA1c: 5.4%

Abbreviations: ACE, angiotensin-converting enzyme; AE, adverse events; BID, twice daily; BMI, body mass index; CR, case report; CS: case series; RCT: randomized controlled trial; DLQI, Dermatology Life Quality Index; HbA1c, glycated haemoglobin; IFG, impaired fasting glucose; iNKT, invariant natural killer T cells; IQR, interquartile range; nbUVB, narrowband ultraviolet B; NR, not reported; NS, not significant; PASI, Psoriasis Area and Severity Index; PUVA, psoralen plus ultraviolet A; T2DM, type 2 diabetes mellitus; TNF-α, tumour necrosis factor alpha; WC = waist circumference; FG = fasting glucose; HOMA-IR = Homeostatic Model Assessment of Insulin Resistance ;LDL = low-density lipoprotein cholesterol; CRP = C-reactive protein; ET = epidermal thickness; Hcy = Homocysteine
